# Infant adoptions in wild bonnet macaques (*Macaca radiata*)

**DOI:** 10.1007/s10329-022-01017-w

**Published:** 2022-09-13

**Authors:** Ashvita Anand, Nagarathna Balakrishna, Mewa Singh, Lynne A. Isbell, Sindhuja Sirigeri, Anushka Saikia, Małgorzata E. Arlet

**Affiliations:** 1 Faculty of Biology, Jagiellonian University, Kraków, Poland; 2 Zoo Outreach Organization, Coimbatore, India; 3 Department of Psychology, University of Mysore, Mysuru, India; 4 Department of Anthropology and Animal Behavior Graduate Group, University of California, Davis, CA USA; 5 Faculty of Biology, Institute of Human Biology and Evolution, Adam Mickiewicz University in Poznań, Poznań, Poland

**Keywords:** Adoption, Age, Bonnet macaque, Mother’s rank, Sex

## Abstract

Though uncommon, adoption of orphaned infants has been observed in both wild and captive non-human primates. In two groups of wild bonnet macaques (*Macaca radiata*), we observed five instances of infants being cared for after they lost their mothers at a pre-weaning age (< 6 months). Orphaned infants had one or more caregivers (juvenile, subadult, and adult female or male) involved in carrying, grooming, hugging, and protecting them. Adoption did not appear to be related to the age/sex class of the infant, or directly to the mother’s rank. Although the dominance rank of the mother of an orphaned infant did not have a direct effect on orphan survivorship, it determined the number of caregivers available to the orphaned infant, and infant survivorship was positively related to the number of caregivers of the orphaned infant. Thus, survivorship was likely a function of the mother’s sociality. Two other infants born to high-ranking mothers were also adopted by more individuals and survived longer than the infants of low-ranking mothers.

**Supplementary Information:**

The online version contains supplementary material available at 10.1007/s10329-022-01017-w.

## Introduction

As mammalian infants must be fed and protected over a long time, their rearing is costly (Silk 1999). Despite that, alloparental care is frequently observed among mammals (Clutton-Brock 2019; Silk 1999), and especially in primates (Clutton-Brock 2019; Kleiman and Malcolm 1981). Potential explanations for alloparenting include kin selection, reciprocal altruism, ‘learning to mother’, i.e., enhancement of the alloparent’s own skills, preexisting social bonds, gaining a future social ally, misplaced reproductive function (i.e., biological programming to care for young animals), increased access to mothers as an alternative mating strategy, or simply attraction towards young infants (Isler and van Schaik 2012; Kerhoas et al. 2016; Maestripieri 1994; Mitani and Watts 1997; Ross and McLarnon 2000). Alloparents are usually females (Silk 1999; Thierry and Anderson 1986) but alloparental care may also be provided by males. Instances of males caring for infants have been observed in catarrhines where male care is otherwise rare, such as in Japanese macaques (*Macaca fuscata*) (Nakamichi et al. 2021). By caring for infants, males may increase their interactions with mothers and their chances of future matings with the mothers, an alternative to direct male–male competition as a mating strategy (*M. fuscata*: Gartland et al. 2020; Nakamichi et al. 2021; *M. sylvanus*: Ménard et al. 2001; *Gorilla beringei*: Rosenbaum et al. 2018).

Adoption is a specific kind of alloparental behavior in which a non-mother gives primary care, such as nursing, carrying, hugging, and protecting, to an orphaned or abandoned infant for an extended period of time (Thierry and Anderson 1986). Various costs, benefits, and motivations related to adoption have been reviewed in detail (Boesch et al. 2010; Hamilton et al. 1982; Hobaiter et al. 2014; Thierry and Anderson 1986; Tokuyama et al. 2021). For example, in chacma baboons (*Papio ursinus*), the costs of adoption for females in terms of interference with direct reproductive potential are likely to be lowest for old menopausal females and females large enough to carry infants but before their first pregnancy (Hamilton et al. 1982). Costs to adult males of adopting their probable offspring may be reduced mating opportunities or reduced care to their other offspring but these costs may be offset by greater probability of orphan survival (Hamilton et al. 1982). As in other alloparental behaviors, the likelihood and expression of behaviors by adoptive individuals may differ depending on the age and sex of the infant, its relationship with the caregiver, and the reproductive stage of the adopting individual (Thierry and Anderson 1986).

According to kin selection theory, individuals will direct positive or altruistic behavior towards individuals to whom they are closely related, even at a cost to themselves (Hamilton 1964). Infant handling can increase the inclusive fitness of the handler by aiding the survival of a related infant, and shortening weaning times and inter-birth intervals of related mothers (*P. cynocephalus*, *P. ursinus*: Silk et al. 2003a, b; *Pan paniscus*: Boose et al. 2018). In cases where adoption occurs in a group of closely related individuals, this behavior may be consistent with kin selection theory, but at the same time it does not exclude other explanations, such as ‘learning to mother’ or acting upon preexisting social bonds. As relatedness in wild populations has been difficult to determine until recently, the frequency of adoptions by relatives is still unclear.

With infant care as a form of reciprocal altruism (not dependent on relatedness), individuals exchange handling behaviors for later infant care or any other social commodity, such as grooming or potential future mating opportunities (e.g., *P. anubis*: Lemasson et al. 2008; *P. ursinus*: Busse and Hamilton 1981). Of all primate species, humans are the most frequently observed to adopt young infants after the death of the primary caregiver (Silk 1987). Frequent adoptions of unrelated individuals with whom adopters have no known social relationship suggest the possibility of reciprocal altruism (Boesch et al. 2010), with payoffs perhaps accruing when the orphan is grown and the adoptive parent is old and in need of care. In non-human primates, caring for old individuals has not been reported, to our knowledge, and adoption is uncommon, but cases have been observed in both catarrhines and platyrrhines (Dunham and Opere 2016), including adoption of unrelated infants, so reciprocal altruism could apply. Boesch et al. (2010) suggested reciprocal altruism as a possible explanation for adoption in Taï chimpanzees (*P. troglodytes verus*), as of 12 adoptions by individuals with known relatedness, seven were by unrelated individuals, and only one case involved the adoptee’s father. One long-term benefit proposed by the authors for the adopting males was that the male infant might grow up to become an ally. However, the adoption of female infants was attributed simply to altruism (Boesch et al. 2010). Similarly, two cases of cross-group adoptions by female bonobos have been observed. One female took care of the adoptee along with her own infant. The other female who adopted an infant was presumed to be post-menopausal (Tokuyama et al. 2021). In other catarrhines, an adult female Angolan black-and-white colobus monkey (*Colobus angolensis*) also adopted an infant from outside her own group and raised it along with her biological infant. Following the loss of her own infant, the female continued to nurse and care for the adoptee (Dunham and Opere 2016). In platyrrhines, Rossi et al. (2020) described the adoption of an orphaned infant black-and-gold howler monkey (*Alouatta caraya*) after being introduced by the researchers to an unrelated adult female in a captive setting. The adoption was successful in that the infant survived into adulthood.

Enhancement of one’s mothering skills, or the ‘learning to mother’ hypothesis, is specific to young, nulliparous females. It suggests that they benefit from gaining experience in maternal behavior while taking care of someone else’s infant (*Chlorocebus pygerythrus*: Meaney et al. 1990; *M. sylvanus*: Paul and Kuester 1996; *P. paniscus*: Boose et al. 2018). In the study of Taï chimpanzees, one juvenile female was among the adopters, although she was also the sister of the orphan (Boesch et al. 2010), so we cannot rule out that adoption also occurred because of kin selection, preexisting social bonds, or the benefits of a future social ally.

As the examples above reveal, adoption has been observed rarely and in primate species with different kinds of social structures, social interactions, and social relationships (Kappeler and van Schaik 2002). Focusing on macaques, adoption by adult females has been documented in captive rhesus (*M. mulatta*) (Deets and Harlow 1974; Ellsworth and Andersen 1999), Japanese (*M. fuscata*) (Fuccillo et al. 1983), and long-tailed (*M. fascicularis*) macaques (Cho et al. 1986), and semi-free-ranging Barbary macaques (*M. sylvanus*) (Paul and Kuester 1996). A more recent study reported adoption of an orphaned infant by her sister in a wild population of Taihangshan macaques *(M. mulatta tcheliensis*, Guo et al. 2022). Interestingly in this case, alloparental care was also provided to the infant by two adult males in the troop. As a genus, macaques are conservative in that they all live in multi-male, multi-female social groups, but different clades of macaques can be classified by differences in social tolerance (Thierry et al. 2000). One might expect adoption to occur in the more socially tolerant clades, but *M. fuscata*, *M. mulatta,* and *M. fascicularis* are in the two least tolerant clades (out of four) (Thierry 2022).

Like other macaques, bonnet macaques (*M. radiata*) predominantly live in multi-male, multi-female groups, in which females are philopatric and males tend to disperse when they reach sexual maturity (Singh et al. 1984). Females form stable and matrilineally heritable dominance ranks (Silk et al. 1981) and are classified as a relatively tolerant species, along with Barbary, toque (*M. sinica*), liontailed (*M. silenus*), and stumptailed (*M. arctoides*) macaques (Thierry 2022). Most female bonnet macaques give birth during the dry season (typically February to May) just before the monsoon season (Rahaman and Parthasarathy 1969; Sinha 2001). After the infant is born, a close mother–infant relationship, while strongest during the first few weeks of life, continues for about 6–10 months (around the time of weaning) (Nathan and Kaufman 1972; Rahaman and Parthasarathy 1969; Simonds 1965; Singh et al. 1980).

Previous studies have indicated the relative absence of alloparenting in bonnet macaques (Rahaman and Parthasarathy 1969; Silk 1999; Simonds 1974), and possible adoption has been reported only once in this species (Singh 2017). Two mothers abandoned their weaned infants at approximately ~ 8 months of age when they immigrated into different groups after the original group fissioned into three sister groups, and the infants were later cared for by other members of the group. However, since no detailed information is available on the type of caregiving behaviors observed ‘adoption’ as such is not confirmed (Singh 2017).

Here, we report five cases of adoption in bonnet macaques, and aim to explore the determinants of adoptions that are successful, i.e., that allow the infant to survive, while also adding to existing records of adoptions in primates.

## Materials and methods

### Study site and subjects

The study was carried out from November 2019 until February 2022 on two habituated bonnet macaque groups (Dam and Eco) at the Thenmala Dam and Ecotourism Recreational Area (8.90 N, 77.10 E) located on the outskirts of Thenmala town in the state of Kerala in southern India. The home ranges of the study groups include small forest patches, three villages, governmental forest offices, and an ecotourism center (Supplementary Fig. 1).

The Dam group consisted of 35 individuals, and the Eco group, 42 individuals (Supplementary Table 1). There are also several non-habituated groups of bonnet macaques in the study area.

All macaques in the two groups were well habituated to human presence and were individually recognizable using natural markings such as facial characteristics (color, scars, wrinkles), relative body size, and nipple size and color. Aside from a few exceptional times when they disappeared into deep forest cover, both Dam and Eco groups were observed throughout their range. Animals were classified as infants when they were under 12 months of age (all were born during the study), juveniles were those aged 1–3 years, and subadults were aged 2–4 years (Simonds 1965). The subjects of this study were all 21 mother–infant pairs. However, by December 2021, only ten infants remained. Whenever we noticed a new infant in a group, we counted it as a birth. We designated an infant as ‘dead’ when we first noticed that it was either dead or no longer present in a group (since young infants cannot survive long by themselves).

### Behavioral observations

Teams of 2–3 observers at a time (six observers in total) recorded behavioral observations 5 days a week during a combination of 15-min focal sampling with scan sampling (social interactions, nearest neighbors, and position in the group), and ad libitum observations (agonistic interactions) at distances of approximately 3–10 m from the monkeys. The observers reached inter-observer reliability in individual recognition, focal sampling, all occurrences sampling, and video decoding prior to commencing data collection (range of Cohen’s kappa 0.91–0.98). The focal sampling order in each group was opportunistically determined by first sighting of an adult female that had not yet been sampled during that particular round, while taking care to balance morning and afternoon sampling. At any given time period, the members of the team observed different individuals and they communicated with each other throughout the day about which individuals had and had not yet been followed. We recorded additional information on the interactions (such as carrying, proximity to other individuals, and aggressive encounters) between five adopted infants and other members of their group through all occurrences sampling using mobile phones or field notebooks so as to capture additional data outside of the 15-min videos used as the primary method of focal sampling the infants.

As there are problems with various behavioral definitions of adoption, Thierry and Anderson (1986), in their exhaustive review of adoption in primates, stated that “we avoid the issue of a priori definitions and describe cases which intuitively suggest adoption” (p. 192). However, we considered an orphaned infant *adopted* when it was given primary care by someone other than its mother more than once per day and continuously until the orphan died or the study ended. Protective behavior alone, as was observed for some adult males against humans, was insufficient since adult males are often protective of group members regardless of their ages (MEA, pers. obs.). Kinship between orphaned infants and their caregivers was not known. We defined *caregiving* as holding, carrying, or grooming the infant by anyone other than the infant’s mother.

Over 23 months, we completed 3723 15-min focals (930.75 h of observations). For five adopted infants, during the period from the infant’s birth until mother’s disappearance, we collected in total 1147 min of focal observations, with a mean ± SD = 229.3 ± 242.2 min per infant, and from the adoption until the infant’s death, we collected in total 1125 min of focal observations, with a mean ± SD = 225.06 ± 218.2 min per infant (TH = 527, BB = 150, NM = 56, SI = 374, SR = 18 min). Only one of the five infants, TH, survived and was still alive as of the end of June 2022.

### Data analysis

Dominance ranks of females were based on 750 recorded dyadic agonistic interactions (Dam: 474, Eco: 276). Agonistic interactions included non-physical threats (e.g., facial displays), approach–avoids (moving away from another who is approaching), supplants (taking the place of another), physical contacts (e.g., biting, tail-pulling, and pushing), and chases (aggressively pursuing another). From these interactions, we constructed dominance matrices for males and females in each study group following Landau’s (1951) Strength of Dominance Hierarchy (h: range 0 (completely egalitarian system) to 1 (a perfect linear order)). Landau’s index was modified by Appleby (1983) where, if two individuals did not interact, they were each given a score of 0.5, and Zumpe and Michael (1986) where they were assigned a score of zero each. Singh et al. (1992, 2003) modified Landau’s index further where the dominance scores were based on the proportion of fights won in dyadic interactions and empty cells were filled by the probability of winning/losing in a dyadic interaction based on the total fights won/lost by the two individuals of the dyadic cell. Further, Singh et al. (2003) converted the dominance ranks from an ordinal scale to an interval scale which indicates not only the dominance ranks but also the intervals between/among ranks.

We used generalized linear mixed models (GLMM) to test for the impact of infant sex and dominance rank of the mother on the duration of caregiving behaviors toward orphaned infants. The dependent variable was the average duration of being held, carried, and groomed per minute from the focal period of 15 min for each infant. Infant identity was considered as a random factor. The impact of infant age on the duration of caregiving behaviors was tested separately using a simple regression model. Results of the GLMM and simple regression model were obtained with STATISTICA 10 (StatSoft Inc. USA) with a significance threshold set at 0.05.

To determine the variables that affected the survivorship of orphaned infants, i.e., maternal characteristics (dominance rank), infant characteristics (age and sex), and number of caregivers, we ran a generalized linear model in RStudio v.1.4.1717 (RStudio Team 2021). Maternal rank was coded as an ordinal variable, sex of infant was coded as a binary categorical variable, and the remaining were treated as continuous numerical variables. We used the glm() function contained in the *stats* package in R for modeling survivorship of orphaned infants as a Poisson distribution. The relationship between the number of caregivers and maternal dominance ranks was determined by logistic regression using the multinom() function within the *stats* package. Results of the Omnibus test to contrast the hypothesized logistic regression model with the intercept-only model were obtained with IBM SPSS v.20 statistical software (IBM 2011). The relationship between rank and percent grooming received by the females who disappeared was determined by logistic regression using the multinom() function within the *stats* package.

## Results

### Dominance ranks of females

Based on the interval score, in the Dam group we classified females as: high rank (2.18–3.24; *N* = 3), middle rank (1.09–2.17; *N* = 11), low rank (0–1.08; *N* = 3), and in the Eco group: high-rank (1.96–2.93; *N* = 1), middle rank (0.98–1.95; *N* = 8) and low rank (0–0.97; *N* = 3) (Supplementary Table 2). Overall* h* (strength of hierarchy) was weak among females in the Dam group (*h* = 0.54), and a bit higher among females in the Eco group (*h* = 0.65).

The dominance ranks of the mothers of the orphaned infants were positively related to the grooming the mothers received from others (regression: *β* = 24.25, *p* = 0.05, *R*^2^ = 0.774) (Supplementary Table 3).

### Interactions between orphans and other group members

On August 18, 2021, in the Eco troop, four infants between the ages of 1 and 5 months lost their mothers due to unknown circumstances. All of these infants appeared healthy at the time they became orphans. On the following day, three of the infants, all males (TR, NM, SI), were observed within a macaque body length near adult males and juvenile females. The fourth, a female (SR), was not seen again. In the second group, Dam, on October 7, three adult females disappeared, one of whom had an infant, BB, who was left behind. These females have not been seen in any of the other groups in the area, and we assume they died or were killed. Characteristics of the orphaned infants are summarized in Supplementary Table 4.

The way in which each orphan was cared for was different. Because adoptions in bonnet macaques are rare, we describe them in detail. Infant SR lost her mother on August 18, 2021, at the age of 4 weeks. Her mother (SP) was a low-ranking female in the Eco group. SR was the youngest infant in the group when her mother went missing. On the first day that her mother disappeared, SR was not seen in the group and was presumed dead/missing. Two days later, however, a subadult female AA was seen carrying her. On two occasions, AA was chased by juvenile and subadult males while she was carrying SR. SR continued to get weaker and was left alone for 5–15 min bouts with no individuals within 2 m, especially in the last few days of her life. SR disappeared from the group a week after being orphaned.

Infant NM lost his mother on August 18, 2021, at 5 months and 10 days of age. His mother NO was a middle-ranking female in the Eco group. On the first day after her disappearance, NM was carried by subadult female LL. Thereafter, he was mostly seen to move around independently. On one occasion, young juvenile male ZI also carried him for a few minutes. NM was not very central in the group, and sometimes was observed with females that usually stayed at the periphery of the group, including PN, whose son, PT, was born 1 day apart from NM. None of those females exhibited any caregiving behaviors, however. He was seen foraging and feeding by himself without engaging in any interactions with any group members until he disappeared from the group on September 13, 2021 at 6 months 5 days of age.

Infant SI lost his mother on August 18, 2021, at the age of 2 months and 1 week. His mother SH was a middle-ranking female in the Eco group. After the mother’s disappearance, SI was first observed making contact calls with adult male SF nearby. Two days later, SI was being carried around by sub-adult female LL. Then, for a few days, SI was mostly by himself, not carried around by other individuals. After that, until mid-September he was mainly carried by sub-adult female LL, but with care also from two other sub-adult females (CD and AA), who approached and picked SI up on a few occasions when he made long contact woos. Then, during the first 2 weeks of October, LL carried SI less, while CD or AA began to carry him more. This was followed by an incident when many adult females suddenly turned aggressive towards AA, chasing, grabbing, and biting her for unknown reasons. Towards the end of October and early November, SI was seen mostly by himself (5-10 m away from all individuals), giving out long contact woos and squeals. He was often seen entering houses and begging for food by himself. AA, CD, and LL only approached and carried him when the group was traveling across their range. Juvenile male NT was seen on two occasions carrying and hugging SI. He was last seen with the group in the middle of the third week of November when he was 4 months 7 days old.

Infant BB lost her mother on October 8, 2021 at the age of 2 months and 2 days. Her mother (BE) was a high-ranking female in the Dam group. BB was the youngest infant in the Dam group when her mother went missing. On that day, BB moved around independently following the group and making long contact woos. On multiple occasions, BB approached adult female ME, who was estimated to be an old parous female in 2016 and had not given birth since. Initially, ME rejected BB when she tried to climb on her or suckle. However, from the second day onwards, ME was seen carrying and holding BB. We also observed a juvenile female MY and adult females YO and KM holding and carrying BB multiple times. On one occasion, an adult female JN also groomed and held her. BB was independent and was often observed moving on her own to feed but usually ME (or sometimes MY) carried her in the presence of danger (a dog or vehicle). BB disappeared in the third week of November when she was 3 months and 12 days old.

Infant TH lost his mother on August 18, 2021, at the age of 3.5 months. His mother (TR) was the highest-ranking female in the Eco group. On the day after his mother disappeared, TH followed BC (the alpha male) that entire day, and BC carried and allowed TH to feed and stay near him. The next day, we were unable to locate the group, but on the following day, TH was carried by a sub-adult female AA for most of the observation time, and he also fed independently. On the fifth day after losing his mother and later, TH was carried by a sub-adult male HD. TH also followed BC, was carried by him, and was allowed to stay and feed around BC and other adult males. After HD emigrated from the Eco to the Dam group in September, TH was carried by juvenile males DB and ZI. Although TH foraged and fed independently on multiple occasions, adult males BC, JK, HN, and HW stayed less than 5 m from him and protected him from dogs, and threatened observers and other humans when they were perceived to approach TH too closely. In the second month, TH was observed to forage and feed on his own in the presence of male juveniles DB or ZI. When the group moved from one location to another, either DB or ZI carried or walked along with him. On multiple occasions, JK was seen giving threats and grabbing TH during feeding bouts on anthropogenic foods when they were at body length. In response, TH would avoid contact or flee. Following one of these aggressive interactions JK was observed grooming TH. TH did not engage in play behavior with other infants and frequently displayed aggressive behavior towards them during feeding or when they approached him. In the third month, on multiple occasions juvenile male HG was also seen close to TH, carrying and hugging him. TH, the lone surviving orphan, remained alive as of June 2022.

We found that the durations of holding, carrying, and grooming orphans were not related to infant sex or mother’s dominance rank (Table 1). Infant age was not related to the duration of any carrying behaviors (hold, carry, groom).

**Table 1:** Summary of generalized linear models to test the effects of dominance rank of the mother and sex of the orphaned infant on duration (min.) of holding (a), carrying (b), and grooming (c)

Variables	SS	*df*	*F*	*p*
Hold				
Constant	0.170	1	58.32	0.08
Mother’s rank	0.020	1	2.23	0.43
Sex of infant	0.010	1	6.52	0.24
Carry				
Constant	0.041	1	116.40	0.06
Mother’s rank	0.048	1	37.01	0.12
Sex of infant	0.010	1	0.89	0.52
Groom				
Constant	1.221	1	79.77	0.07
Mother’s rank	2.078	1	20.27	0.16
Sex of infant	0.463	1	3.75	0.30

Analysis revealed that the survivorship of orphaned infants depended solely on the number of caregivers and was independent of the mother’s dominance rank per se, and the age and sex of the infant (Table 2). Every additional caregiver increased the survivorship of an orphaned infant by about 1.5 times (exp (β_Caregivers_)) over the average duration of orphaned infant survivorship (Table 2). Although the dominance rank of the mother of an orphaned infant did not have a direct effect on orphan survivorship, it determined the number of caregivers available to the orphaned infant (logistic regression; ${\chi }_{Caretakers}^{2}$ = 9.503, df = 2, *p* = 0.009; ${R}_{Cox-Snell}^{2}$ = 0.851).

**Table 2:** Summary of generalized linear model to test the effects of dominance rank of the mother, sex of the orphaned infant, age of the orphaned infant (days), and number of caregivers of the orphaned infant on duration of orphaned infant survival (days). Duration of orphaned infant survival is modeled as a Poisson distribution with logarithmic link function

Variables	Factors	β	SE	*p*
Orphaned infant survival ~ Mother’s dominance rank + Sex of orphaned infant + Age of orphaned infant + Caregivers of orphaned infant				
*Constant*	–	2.077	0.386	< 0.001
Mother’s dominance rank	High (ref.)			
	Low	− 0.536	0.466	0.250
	Medium	0.336	0.336	0.317
Sex of infant	Male (ref.)			
	Female	0.002	0.169	0.988
Age of orphaned infant	–	− 0.00	0.009	0.988
Number of caregivers	–	0.403	0.060	< 0.0001
*Model fit*		Omnibus test		
Log likelihood	27.074	Likelihood ratio *χ*^2^		173.040
		*df*		4
		*p*		< 0.0001

## Discussion

Infant adoption has been observed in a variety of animal species but has not been often described. Here, we have presented five cases of adoption in bonnet macaques—by adult males and adult and subadult females, and juveniles of both sexes—to supplement the two possible cases that have already been reported in this species (Singh 2017). Note that the events reported were rather short. We acknowledge that it may be debatable whether they fall under true adoption. Infant survival was dependent on the number of group members involved in caring for the orphan, but not its sex, age, or mother’s rank per se. Mother’s rank was, however, found to be a function of the mother’s sociality in that high-ranking females were groomed more than middle- and low-ranking females before they died, and their orphaned offspring had more caregivers, and survived longer, than the orphans of middle- and low-ranking females. Orphans of high-ranking females were also allowed to feed around adult males and tolerated well. The surviving orphan stands out because he had the greatest number of caregivers, including the alpha male, who provided care only to him, and his mother was the highest-ranking female. In baboons, mother’s sociality is also positively associated with infant survival (Silk et al. 2003a). Thus, in our study, although a direct effect of the mother’s rank was not observed, being high-ranking increased the number of members in a mother’s social network. This in turn increased the number of infant caregivers following the mother’s demise. Age and sex seem less important in determining survival because the survivor was not the oldest of the orphans nor did other males survive.

While mother’s rank had an indirect effect on orphan survivorship, it is possible that mother’s health also contributed indirectly. Higher-ranking individuals often have better access to foods than lower-ranking individuals, for instance (Barton and Whiten 1993; Murray et al. 2006; Vries et al. 2020). Higher-ranking individuals may also experience less stress, as suggested by lower levels of cortisol (Abbott et al. 2003; Cheney and Seyfarth 2009). It is possible that health benefits of high-ranking mothers translated to infants that were healthier than infants of lower-ranking mothers and thus more equipped to survive as orphans than their subordinate counterparts (Majolo et al. 2012; Silk et al. 2003a). However, differences in health among the orphans in our study were not visibly apparent.

In our study, macaques of multiple ages and both sexes served as caregivers, suggesting that there may be multiple adaptive explanations for their behavior. Because genetic data were not available, we could not confirm that kin selection was involved in caregiving. We can rule out the latter explanation for the alpha male’s caregiving because the mother was dead when he served as a caregiver. Thus, it is likely that his caregiving efforts were driven by his kinship with the orphan.

Nowadays, there is a broad consensus that kin selection underlies altruistic behavior among genetic relatives but in contrast, there is much less agreement about the processes that underlie beneficent behavior toward nonrelatives, and, since it is even unlikely that reciprocal altruism exists in most animals, including monkeys (Silk 2007), we cannot regard the possibility of infant adoptions as reciprocal altruism.

Support for the ‘learning to mother’ hypothesis depends on the involvement of immature females (juveniles and subadults) in caregiving. In fact, immature females were the most numerous caregivers. The females carried, groomed, and facilitated movement of the infants through the range, suggesting that they could be ‘learning to mother’ (*C. pygerythrus*: Meaney et al. 1990; *M. sylvanus*: Paul and Kuester 1996; *P. paniscus*: Boose et al 2018). In the Eco group specifically, the subadult females that were involved in the caregiving process were close to maturity and were likely to conceive their first infants within the next one or two breeding seasons.

Finally, we cannot rule out pre-existing social bonds as a proximate explanation for the adoptions. The finding that orphan survivorship was positively associated with the number of caregivers and the mother’s social network suggests that adoptions were facilitated by pre-existing relationships. This is similar to the findings of Silk et al. (2003a) from wild yellow baboons (*P. cynocephalus*). In their 16-year study, they found that adult female sociality, as measured by proximity to other adults, grooming other adults, and being groomed by other adults, was positively correlated with the survivorship of infants. Silk and colleagues also found that the relationship between sociality and infant survivorship was independent of dominance rank.

Adoption may not always be adaptive, but helpers may gain some benefits from providing care, such as enhancing their inclusive fitness or learning new skills that improve the chances of being successful primiparous mothers. While there may be several forces driving adoption depending on the adopter’s age, sex, and relationship to the orphan, it is clear that adoption in macaques is a phenomenon that requires more investigation, particularly with genetic data, so we may better understand the role that kin selection plays, along with other factors, such as social tolerance and learning to mother, in the process of adoption.

## Supplementary Information

Below is the link to the electronic supplementary material.

Supplementary file1 (PDF 220 KB)

## Data Availability

The datasets analyzed during the current study are available from the corresponding author on reasonable request.
