# Supplementary material for: Infant adoptions in wild bonnet macaques (Macaca radiata)
Source: Primates. 2022 Sep 13;63(6):627–35. doi: 10.1007/s10329-022-01017-w (PMC9646593; doi:10.1007/s10329-022-01017-w)
Supplement: Supplementary file 1 — Supplementary file1 (PDF 220 KB) [file 10329_2022_1017_MOESM1_ESM.pdf]

Figure 1. Map of the Thenmala site with home ranges of study groups.

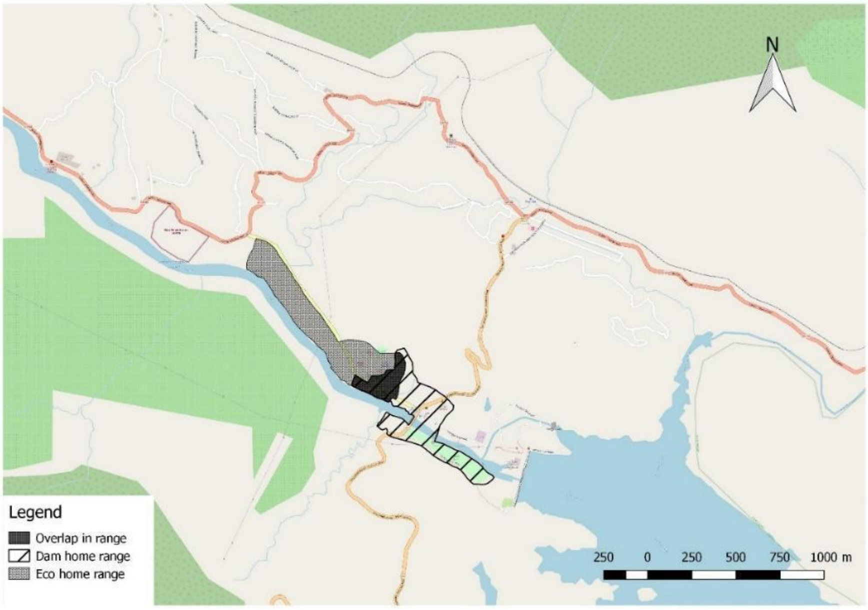

Table 1. Composition of bonnet macaque study groups in 2021 (when adoption was observed) in Thenmala, Southern India.

	Dam	Eco
Group size	35	42
Adult females	10	7
Adult males	6	10
Subadult females	2	3
Subadult males	2	2
Juveniles (both sexes)	10	11
Infant females	3	1
Infant males	2	8

Table 2. Dominance scores and classification of female ranks

Dam group				Eco group			
Individual ID	Ordinal score	Interval score	Rank class	Individual ID	Ordinal score	Interval score	Rank class
BL	.95	3.24	HR	TR*	.95	2.92	HR
BE*	.85	2.61	HR	LE	.73	1.93	MR
YO	.79	2.39	HR	SH*	.70	1.83	MR
JN	.69	2.06	MR	SY	.63	1.63	MR
ME	.68	2.03	MR	ZM	.58	1.51	MR
ML	.65	1.94	MR	NO*	.51	1.32	MR
KL	.57	1.74	MR	SA	.48	1.24	MR
KM	.55	1.70	MR	PY	.43	1.11	MR
FN	.54	1.67	MR	BV	.43	1.11	MR
KO	.51	1.60	MR	PN	.28	0.73	LR
SC	.47	1.50	MR	BT	.18	0.39	LR
MZ	.44	1.41	MR	SP*	.10	0	LR
AS	.35	1.19	MR				
TS	.35	1.17	MR				
MR	.24	0.87	LR				
MI	.15	0.52	LR				
BV	.06	0	LR				

LR – Low rank; MR – Middle rank; HR – High rank
*Females who disappeared, leaving their infants as orphans

Table 3. Ranks and grooming scores of females that disappeared

Orphan ID	Mother ID	Rank	Grooming given	Grooming received	% grooming received
TH	TR	High	3	47	94
BB	BE	High	67	115.5	63
NM	NO	Middle	74	90.5	55
SI	SH	Middle	22	25	53.5
SR	SP	Low	24.5	10.5	30

Table 4. Characteristics of orphaned infants from two groups (August - December 2021)

Infant	Sex	Group	Mother's rank	Date orphaned	Age when orphaned	# of caregivers	Date of disappearance	Age when disappeared	Days survived after being orphaned
SR	F	Eco	Low	18/08/2021	1m	1	25/08/2021	1m 7d	7
NM	M	Eco	Middle	18/08/2021	5m 10d	2	13/09/2021	6m 5d	25
SI	M	Eco	Middle	18/08/2021	2m 7d	4	18/11/2021	4m 7d	40
BB	F	Dam	High	08/10/2021	2m 2d	5	18/11/2021	3m 12d	60
TH	M	Eco	High	18/08/2021	3m 15d	7	NA	NA	NA
